# Mechanism of action of Butein in cutaneous squamous cell carcinoma through regulation of the TWEAK-FN14 signaling pathway

**DOI:** 10.3389/fonc.2025.1725848

**Published:** 2025-12-11

**Authors:** Qinyi Dong, Zijian Zhang, Siying Li, Xinman Wang, Han Zhang, Jiahao Bai, Kaili Zheng, Lili Liang

**Affiliations:** 1 The Third Clinical College, Shanxi University of Chinese Medicine, Taiyuan, China; 2 Fifth Clinical Medical College, Shanxi Medical University, Taiyuan, China; 3 Department of Plastic and Aesthetic Surgery, Shanxi Provincial People’s Hospital Affiliated to Shanxi Medical University, Taiyuan, China; 4 Department of Dermatology, Fenyang Hospital of Shanxi Province, Fenyang, China

**Keywords:** Butein, cutaneous squamous cell carcinoma, TWEAK-Fn14 signaling, tumor microenvironment, anti-tumor mechanism

## Abstract

**Background:**

The incidence of cutaneous squamous cell carcinoma (cSCC) continues to rise, while current therapeutic approaches remain limited in efficacy for patients with advanced disease. The natural polyphenol Butein has demonstrated antitumor activity in various malignancies; however, its role and underlying mechanisms in cSCC remain unclear. Our previous study revealed that the TWEAK-FN14 axis promotes cSCC proliferation by activating the NF-κB and STAT3 pathways, whereas Butein can inhibit both pathways, suggesting that it may exert anti-cSCC effects by targeting the TWEAK-FN14 axis.

**Objective:**

To investigate whether Butein affects cSCC growth by modulating the TWEAK-FN14 signaling pathway and its tumor microenvironment, and to elucidate the underlying molecular mechanisms.

**Results:**

Molecular docking predicted that Butein exhibits strong binding affinity with TWEAK, FN14, cIAP1, and TRAF1/2 proteins, with binding energies ranging from −5.8 to −6.9 kcal/mol.*In vitro* experiments demonstrated that Butein inhibited the proliferation and migration of human cSCC cell line A431 with an IC_50_ of 43 μM and induced dose-dependent apoptosis. In a nude mouse xenograft model, treatment with Butein at 10, 20, and 40 mg/kg reduced tumor volume by 39.21%, 63.44%, and 79.05%, respectively, without affecting body weight. Mechanistic studies revealed that Butein markedly downregulated the protein and mRNA expression of TWEAK, FN14, and TRAF1/2 in tumor tissue, and decreased serum levels of NF-κB-related inflammatory factors, including IL-1β, IL-6, IFN-γ, and TNF-α.

**Conclusion:**

Butein effectively suppresses cSCC growth by directly binding to and inhibiting key proteins in the TWEAK-FN14 signaling pathway, thereby coordinately modulating the downstream inflammatory microenvironment. This study provides mechanistic insights and experimental evidence supporting Butein as a potential therapeutic candidate for cSCC.

## 1 Introduction

Cutaneous squamous cell carcinoma (cSCC) is a type of keratinocyte carcinoma and ranks second among skin cancers, following basal cell carcinoma (1). In the United States, approximately 1.1 million new cases of cSCC are diagnosed each year (2). In China, the prevalence of cSCC has markedly increased over the past 30 years and is projected to continue rising over the next decade at a rate exceeding the global average (3). Most cases of cSCC can be cured through complete excision, and patients generally have a favorable prognosis. However, an estimated 3-7% of patients develop metastasis, particularly those with aggressive variants of cSCC, which tend to grow rapidly and metastasize frequently, thereby significantly increasing morbidity and mortality (4). Therefore, the development of new diagnostic and therapeutic strategies requires a deeper understanding of the molecular mechanisms underlying this disease.

Butein (3,4,2′,4′-tetrahydroxychalcone) is a polyphenolic compound isolated from the stem bark of the cashew tree, the Genus Sophora, the lacquer tree, and Dalbergia heartwood, among other traditional medicinal plants (5). Studies have shown that Butein possesses multiple biological activities, including anti-inflammatory (6), antitumor (7, 8), antioxidant (9), and anti-angiogenic effects (10), and it exerts preventive and therapeutic effects on various diseases such as inflammatory disorders, tumors, cardiovascular diseases, and diabetes (11). In recent years, numerous studies have confirmed that Butein can inhibit the growth, proliferation, metastasis, and angiogenesis of tumor cells and promote their apoptosis through multiple targets and signaling pathways. It has demonstrated inhibitory effects on a variety of cancers, including oral squamous cell carcinoma (8), lung cancer (12), liver cancer (13), colorectal cancer (14), breast cancer (15), ovarian cancer (7), and stomach cancer, indicating that Butein is a highly promising antitumor drug. Studies have shown that Butein can promote the interaction between the E3 ligase FBW7 and MCL-1, thereby enhancing MCL-1 Ub-K48 ubiquitination and degradation. Butein exhibits potent anti-inflammatory and antitumor activities in oral squamous cell carcinoma and liver cancer by inhibiting IκBα phosphorylation, p65 nuclear translocation, and the expression of downstream inflammatory factors (IL-6 and TNF-α) (8, 13). In ovarian cancer and non-small cell lung cancer, Butein suppresses STAT3 phosphorylation, downregulates PD-L1 expression, and blocks tumor immune evasion (7, 12). Results from both *in vitro* cell experiments and *in vivo* xenograft models demonstrate that Butein possesses significant antitumor activity and good tolerability, suggesting its potential as a promising therapeutic agent for cSCC (8). However, the detailed mechanisms underlying its inhibitory effects on cSCC require further investigation.

Although Butein has been shown to inhibit tumor growth in oral squamous cell carcinoma (OSCC) through FBW7-mediated ubiquitination and degradation of MCL-1, several limitations remain. First, significant differences exist between skin and oral squamous cell carcinomas in terms of ultraviolet exposure and immune microenvironmental regulation (such as IL-1β/IFN-γ signaling), and direct evidence of Butein’s effects in cSCC is entirely lacking (3). Second, most existing studies remain at the level of phenotypic observation and have not elucidated how Butein penetrates the skin barrier or interacts with stromal cells (such as fibroblasts and keratinocytes) within the tumor microenvironment. Finally, its potential to modulate immune regulatory pathways, such as the TWEAK-FN14 axis, has not been systematically evaluated. Therefore, the uniqueness of this study lies in its first to integration of the multi-pathway inhibitory properties of Butein with the immune-inflammatory driving mechanism of the TWEAK-FN14 axis in cSCC, thereby filling the mechanistic gap across the “natural product-skin tumor-immune microenvironment” triad.Therefore, Butein may serve as a potential traditional Chinese medicine monomer for the treatment of cutaneous squamous cell carcinoma (cSCC).

Our research group previously found that the TWEAK/FN14 axis-mediated NF-κB signaling pathway plays a crucial role in the proliferation of cSCC (16). Both tumor necrosis factor-like weak inducer of apoptosis (TWEAK) and its receptor FN14 are highly expressed in cSCC, which enhances the cytoplasmic distribution of cIAP1 and the ubiquitination of RIP1, accompanied by the activation of the canonical NF-κB signaling pathway. Meanwhile, TWEAK upregulates the protein expression of tumor necrosis factor-related apoptosis-inducing ligand receptor 3 (TRAIL-R3) and TRAIL-R4. Knockdown of FN14, or inhibition of cIAP1 and TRAIL-R3, can block the TWEAK-induced promotion of cell proliferation (15). Therefore, our previous studies demonstrated that the TWEAK-FN14 axis promotes cSCC proliferation through activation of NF-κB and STAT3, both of which are known targets inhibited by Butein.This “dual regulatory node” characteristic suggests that Butein may exert a synergistic inhibitory effect through the TWEAK-FN14→NF-κB/STAT3 cascade pathway; however, no study has yet directly validated this hypothesis. Therefore, whether Butein influences cSCC via the TWEAK/FN14 axis remains to be further investigated.

Based on the above evidence, we propose an innovative “Butein-TWEAK-FN14-tumor microenvironment” triadic hypothesis: first, Butein binds stably to the TWEAK/FN14 protein interface through hydrogen bonding and hydrophobic interactions, thereby blocking ligand-receptor interactions; second, by suppressing downstream signaling, it reduces TRAF1/2 recruitment and inhibits cIAP1-mediated RIP1 ubiquitination, leading to the coordinated inhibition of both NF-κB and STAT3 pathways; finally, it remodels the microenvironment by decreasing key “inflammation-to-cancer transition” factors such as IL-1β and IFN-γ, thereby reversing the immunosuppressive microenvironment. This mechanism not only elucidates the high efficacy of Butein in cSCC but also reveals its multidimensional regulatory advantages in immunity, metabolism, and proliferation, distinguishing it from conventional chemotherapeutic agents such as cisplatin, and providing a precise mechanistic basis for subsequent clinical translation.

## 2 Materials and methods

### 2.1 Molecular docking analysis

To explore the potential interactions between Butein and proteins in the TWEAK-FN14 pathway, a preliminary computational chemistry screening was conducted. The 3D structures of TWEAK, FN14, cIAP1, TRAF1, and TRAF2 proteins were obtained from the Protein Data Bank (www.rcsb.org/pdb/home/home.do) (17). The 3D structure of the compound Butein was retrieved from PubChem (https://www.ncbi.nlm.nih.gov/pccompound) (18). The molecular docking process included protein and ligand preparation, construction of the Docking grid, and execution of compound docking. Following the Schrodinger Glide docking protocol, molecular docking simulations were performed using AutoDockTools-1.5.7 software (19). The optimal docking poses were selected based on hydrogen bonding, electrostatic, and hydrophobic interactions within the ligand-receptor complexes.Grids were generated centered on the catalytic or binding sites of each protein, with an Inner box radius of 12 Å and a van der Waals radius scaling factor of 0.8. A binding energy threshold of ≤ −6.0 kcal/mol was set as the preliminary screening criterion, and the binding energy values (kcal/mol) were used to evaluate the docking results. PyMOL software was employed to visualize hydrogen bond interactions, binding affinities, interacting amino acid residues, and the 3D structures of the ligand-receptor complexes (20). This study was designed as a “mechanistic exploratory experiment,” in which the docking results served as a “hypothesis-generating tool” for subsequent Western blot and immunofluorescence (IF) validation. No redocking RMSD or MD simulation validation was performed. We acknowledge this as a limitation of the present study and plan to conduct experimental binding kinetics measurements using isothermal titration calorimetry (ITC) and surface plasmon resonance (SPR) in future work.

### 2.2 Butein-related gene enrichment analysis

We predicted proteins that bind to Butein using the STRING website (https://string-db.org/). Gene Ontology (GO) Enrichment analysis and Kyoto Encyclopedia of Genes and Genomes (KEGG) pathway analysis were performed on Butein-related genes (21).

### 2.3 Experimental cells

Human cutaneous squamous cell carcinoma cells A431 were purchased from Wuhan Punosai Life Science and Technology Co., Ltd. (Hubei, China). They were cultured in DMEM (Sigma-Aldrich, St. Louis, MO, USA) supplemented with 10% fetal bovine serum and 1% penicillin-streptomycin, and maintained at 37°C in a humidified incubator containing 5% CO_2_.

### 2.4 Laboratory animals

Twenty-eight 3-week-old female Balb/c-nu mice with a mouse body weight of 10–13 g were obtained from SPF (Beijing) Biotechnology Co., Ltd. (Beijing, China). All experiments were conducted in accordance with the Guide for the Care and Use of Laboratory Animals and approved by the Medical Ethics Committee of Fenyang Hospital of Shanxi Province (approval No. 2023023, dated April 28, 2023). The study was carried out following the ARRIVE guidelines. The animals were housed in a pathogen-free facility at 24 ± 2°C, under a 12-hour light/dark cycle, with a relative humidity of 50 ± 5%, and had free access to food and water. Humane endpoint criteria were clearly defined: mice were euthanized when tumor volume exceeded 1500 mm³, mouse body weight loss was greater than 15%, or signs of distress (such as hunching or drowsiness) were observed. Randomization was performed using an Excel random number generator, and investigators were blinded to group allocation during tumor measurement. The power calculation method is described in detail in Section 2.8 (effect size = 0.5, α= 0.05, power = 80%, resulting in n= 6).

### 2.5 Cell viability assay

Cell viability was determined using the Cell Counting Kit-8 (CCK-8; Dojindo, Kumamoto, Japan) according to the manufacturer’s instructions. The treated cells in 96-well plates were washed with PBS and replaced with fresh culture medium containing 10% CCK-8 reagent. The plates were then incubated at 37°C for an additional 1.5 h. The absorbance of the medium, reflecting the staining intensity, was measured at 450 nm using a microplate spectrophotometer (Epoch, BioTek, USA).

### 2.6 Assessment of cell migration ability

Cells were seeded into 6-well plates. Once confluence was reached, a scratch was created across the cell monolayer using a 100 μL pipette tip, and the cells were incubated in serum-free medium. Images of the cells were captured at 0, 24, and 48 h to evaluate migration ability.

### 2.7 Analysis of apoptosis

Apoptosis was evaluated using an Annexin V-FITC Apoptosis Detection Kit (BestBio Biotechnologies, Shanghai, China). A431 cells treated with Butein were first digested with 0.25% trypsin and then resuspended in washing buffer. The collected cell suspension was used for subsequent experiments. The cells were washed three times with ice-cold phosphate-buffered saline (PBS) and immediately fixed and resuspended in an appropriate volume of binding buffer. After fixation, 5 μL of Annexin V-fluorescein isothiocyanate (FITC) and 5 μL of propidium iodide (PI) were added to the cells, followed by incubation in the dark for 15 minutes. Apoptosis was then analyzed by flow cytometry (BD Biosciences, San Jose, CA, United States).

### 2.8 Construction of the cSCC model

After a 7-day acclimatization period, mice were subcutaneously inoculated with 1×10^5^ A431 cells suspended in 100 μL of PBS. Using a random number table, the nude mice were randomly assigned to four groups (n = 6). When the tumors became palpable (day 13), the mice received intraperitoneal injections of Butein. Butein was purchased from Shanghai Yuanye Bio-Technology Co., Ltd. (Shanghai, China) and prepared in PBS containing 5% DMSO and 10% Solutol HS-15 to ensure solubility and stability. The low-, medium-, and high-dose groups were administered 10 mg/kg, 20 mg/kg, and 40 mg/kg of Butein, respectively, diluted in 300 μL of PBS. Mice in the control group received an equivalent volume of normal saline. Injections were performed once daily for two consecutive weeks. Based on the results of the cell experiments, the *in vitro* IC_50_ of A431 cells was determined to be 43 μmol/L (approximately 12.8 μg/mL). According to the body surface area conversion formula (mouse dose = human clinical dose × 12.3) and considering an intraperitoneal bioavailability of approximately 40%, the effective *in vivo* dose was estimated to be 8–15 mg/kg.To cover the therapeutic window and assess the dose-response relationship, three groups were established with doses of 10 mg/kg (equivalent low dose), 20 mg/kg (medium dose), and 40 mg/kg (high dose). Validation using similar models showed that Wang et al. (8) demonstrated in an oral squamous cell carcinoma xenograft model that 10 mg/kg inhibited tumor growth by 40% without toxicity, while Zhou et al. (13) reported that 20 mg/kg represented the optimal effective dose in a liver cancer model. Given the local drug accumulation characteristics of skin tumors, a better therapeutic effect was anticipated at equivalent doses. The sample size was calculated based on previous studies. Considering a significance level of 0.05 [95% confidence interval (α= 0.05)], a power of 80%, and an effect size of 0.5, power analysis estimated that five animals per group were required.In addition, considering a 20% dropout rate, six animals per group (a total of 24 mice) were deemed optimal.

### 2.9 Measurement of tumor volume and serum extraction

The length and width of established tumors were measured using a digital caliper, and tumor growth was recorded every two days. Tumor volume was calculated using the following formula: tumor volume (cm³) = length × (width)² × 0.5. On day 10, mice were anesthetized with the inhalation anesthetic isoflurane, and whole blood was collected via retro-orbital bleeding. The blood samples were allowed to clot at room temperature and then centrifuged at 3500 rpm for 10 minutes. The obtained serum was stored in 1.5 mL centrifuge tubes until further use.

### 2.10 Immunofluorescence staining

Tumor tissue sections were blocked with 1% BSA and incubated overnight at 4 °C with antibodies against TWEAK, FN14, cIAP1, RIP1, and Ki67. After counterstaining with DAPI and mounting, the sections were examined under a fluorescence microscope.

### 2.11 Western blot

Tumor tissues were collected from mice and lysed using RIPA lysis buffer containing protease inhibitors (Cat. No. PC101; Shanghai YaMei Biotechnology Co., Ltd.). Protein concentrations were determined with a BCA Protein Assay Kit (Cat. No. ZJ102; Shanghai YaMei Biotechnology Co., Ltd.). Protein samples (10 μg per lane) were separated by SDS-PAGE on 10-15% gels and subsequently transferred onto nitrocellulose membranes. The membranes were blocked with 5% nonfat milk at room temperature for 2 h and then incubated overnight at 4 °C with the following specific primary antibodies: TWEAK (1:600; Cat. No. AY4426; Abways Technology, Shanghai, China), FN14 (1:1500; Cat. No. CY5798; Abways Technology), cIAP-1 (1:1000; Cat. No. DF6167; Affinity Biosciences Ltd.), RIP1 (UQCRFS1 Ab; 1:1500; Cat. No. CY8295; Abways Technology), and TRAIL-R3 (1:750; Cat. No. 24622-1-AP; ProteinTech Group, Inc.).NF-κB (1:500; Cat. No. ab32042; Abcam). The membranes were then incubated with HRP-conjugated goat anti-rabbit IgG H&L secondary antibody (1:3,000; Cat. No. ab6721; Abcam) at room temperature for 1 hour. The membranes were washed three times with TBS containing 0.05% Tween-20 and visualized using the BeyoECL Star kit (Cat. No. P0018AS).

### 2.12 Real-time quantitative polymerase chain reaction analysis

Total RNA was extracted from Tumor tissue using the Total RNA Extraction Kit (Seven Innovation (Beijing) Biological Technology Co., Ltd., Beijing, China), and its purity was determined with a Bioteke ND5000 spectrophotometer (Wuxi, China). Complementary DNA (cDNA) was synthesized using the All-In-One 5× RT MasterMix (Applied Biological Materials, Vancouver, Canada), and quantitative real-time PCR was performed on a Bio-Rad CFX96 real-time detection system using the BlasTaq 2× qPCR MasterMix (Applied Biological Materials, Canada). The PCR amplification conditions were as follows: Stage 1, 95°C for 3 minutes; Stage 2, 40 cycles at 95°C for 15 seconds and 60°C for 1 minute; Stage 3, 95°C for 15 seconds, 60°C for 60 seconds, and 95 °C for 15 seconds. The primers used for RT-PCR were synthesized by Sangon Biotech (Shanghai) Co., Ltd. and are listed in **Supplementary Table S1**.

### 2.13 ELISA assay

Mouse serum samples were collected and stored at −80°C. According to the manufacturer’s instructions, ELISA kits were used to determine the expression levels of IL-1β (Cat. No.: MM-0040M2), IL-6 (Cat. No.: MM-0163M2), IFN-γ (Cat. No.: MM-0182M2), and TNF-α (Cat. No.: MM-0132M1) in mouse serum. Given that the classical function of the TWEAK-FN14 axis is to activate NF-κB and drive systemic inflammatory responses, this study prioritized the measurement of four representative serum cytokines downstream of NF-κB: IL-1β (Cat. No.: MM-0040M2), IL-6 (Cat. No.: MM-0163M2), IFN-γ (Cat. No.: MM-0182M2), and TNF-α (Cat. No.: MM-0132M1). Quantification was performed using ELISA kits. It should be noted that this assay reflects only the level of systemic inflammation in circulation and cannot be directly equated with the cellular composition, spatial distribution, or local cell-cell interactions within the tumor microenvironment (TME). A comprehensive characterization of the TME requires the integration of *in situ* immunophenotypic analysis and single-cell technologies. This represents a limitation of the present study and a focus for future work.

### 2.14 Statistical analysis

All data were analyzed using SPSS 26.0 software and are presented as mean ± standard deviation. Data were evaluated by one-way ANOVA or two-way ANOVA, followed by least significant difference (LSD) or Dunnett’s *post hoc* tests for multiple comparisons. Differences with p < 0.05 or p < 0.01 were considered statistically significant.

### 2.15 Reproducibility and ample size criteria

All *in vitro* experiments were performed with three independent biological replicates, each containing three technical replicates. Sample sizes were determined according to standard practices in cell biology research: for the CCK-8 assay, each concentration point included n = 6 (96-well plate layout); for the scratch assay, each concentration group included n = 3, with three fields of view per scratch; for flow cytometry, 10,000 cells were collected per sample, with three replicates; and for western blot quantification, three independent cell lysate samples were used.This design ensured a statistical power of ≥80% at an effect size of 0.5 and α = 0.05. Each biological replicate contained three technical replicates; for example, in the CCK-8 assay, six replicate wells were set for each concentration group (derived from the same cell suspension), with three wells used for mean calculation and the remaining three reserved as backups. The sample size determination was based on *a priori* power analysis using G*Power 3.1 software, with an effect size of 0.5, a significance level of α = 0.05, and a statistical power of 1 − β = 0.80, yielding a minimum required sample size of n = 5 per group. Considering a 15% attrition rate in cell-based experiments, the final sample size was set at n = 6 for CCK-8 assays and n = 3 for scratch or western blot experiments. Error bars in all figures represent the standard deviation (SD), reflecting the variability among biological replicates.The quantitative bar graph of the western blot bands was generated based on three independent grayscale scanning datasets.

## 3 Results

### 3.1 Enrichment analysis of Butein-related genes

**Figure 1:**
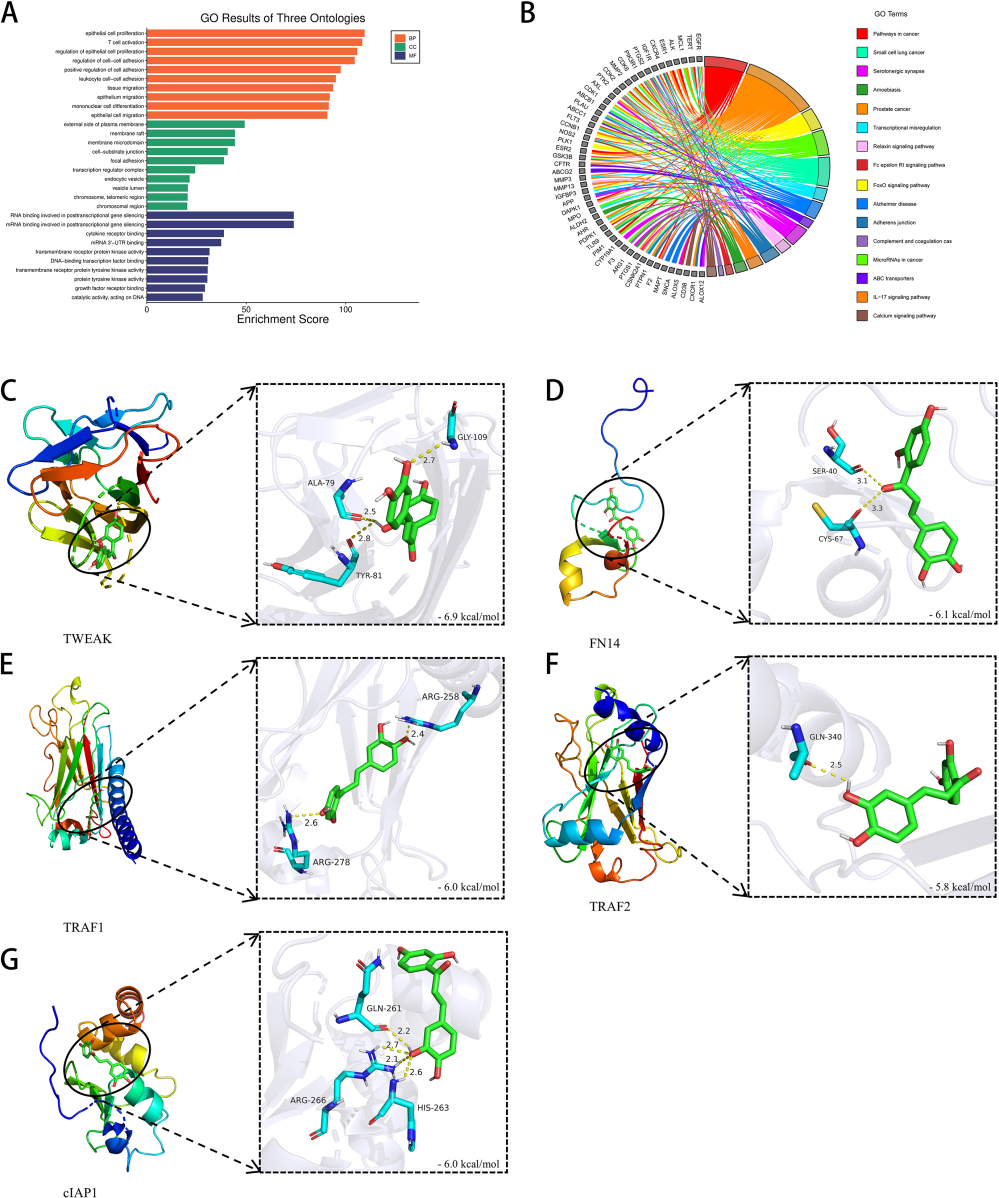
Preliminary screening of Butein-related genes by enrichment analysis and molecular docking. **(A, B)** KEGG/GO analysis revealed that Butein-binding proteins were significantly enriched in cancer-related pathways. Bioinformatics prediction was performed using the DAVID tool based on 104 experimentally verified interacting proteins from the STRING database (v11.5), with a p-value <0.05. **(C–G)** Molecular docking identified the top five proteins with the highest binding affinity for Butein, with the following binding energies: FN14 (−6.1) > cIAP1 (−6.0) = TRAF1 (−6.0) > TRAF2 (−5.8). Note: This molecular docking analysis did not include re-docking validation (RMSD) or molecular dynamics simulation and represents only preliminary screening results.

To investigate the potential mechanisms by which Butein participates in tumorigenesis, we identified Butein-binding proteins and Butein-related genes for pathway Enrichment analysis. Using the STRING tool, a total of 104 Butein-binding proteins were identified, supported by experimental and co-expression evidence. KEGG analysis revealed that these Butein-binding proteins are mainly involved in pathways such as ‘Pathways in cancer’, ‘Prostate cancer’, ‘FoxO signaling pathway’, ‘MicroRNAs in cancer’, ‘Small cell lung cancer’, ‘Transcriptional misregulation’, ‘Alzheimer’s disease’, and ‘ABC transporters’ (**Figures 1A, B**).

### 3.2 Strong binding affinity of Butein with proteins in the TWEAK-FN14 signaling pathway

To further explore the binding capacity of Butein with proteins in the TWEAK-FN14 signaling pathway, molecular docking simulations were performed to determine the potential interactions between Butein and TWEAK, FN14, cIAP1, TRAF1, and TRAF2. The results showed that Butein exhibited strong binding affinities with the aforementioned proteins. The strongest binding energy was observed between Butein and TWEAK at −6.9 kcal/mol, followed by FN14 at −6.1 kcal/mol, cIAP1 at −6.0 kcal/mol, TRAF1 at −6.0 kcal/mol, and TRAF2 at −5.8 kcal/mol (**Figures 1C–G**). These findings suggest that Butein may have favorable interaction capabilities with proteins in the TWEAK-FN14 signaling pathway.

### 3.3 Butein inhibits the proliferation and invasion of A431 cells and promotes apoptosis

**Figure 2:**
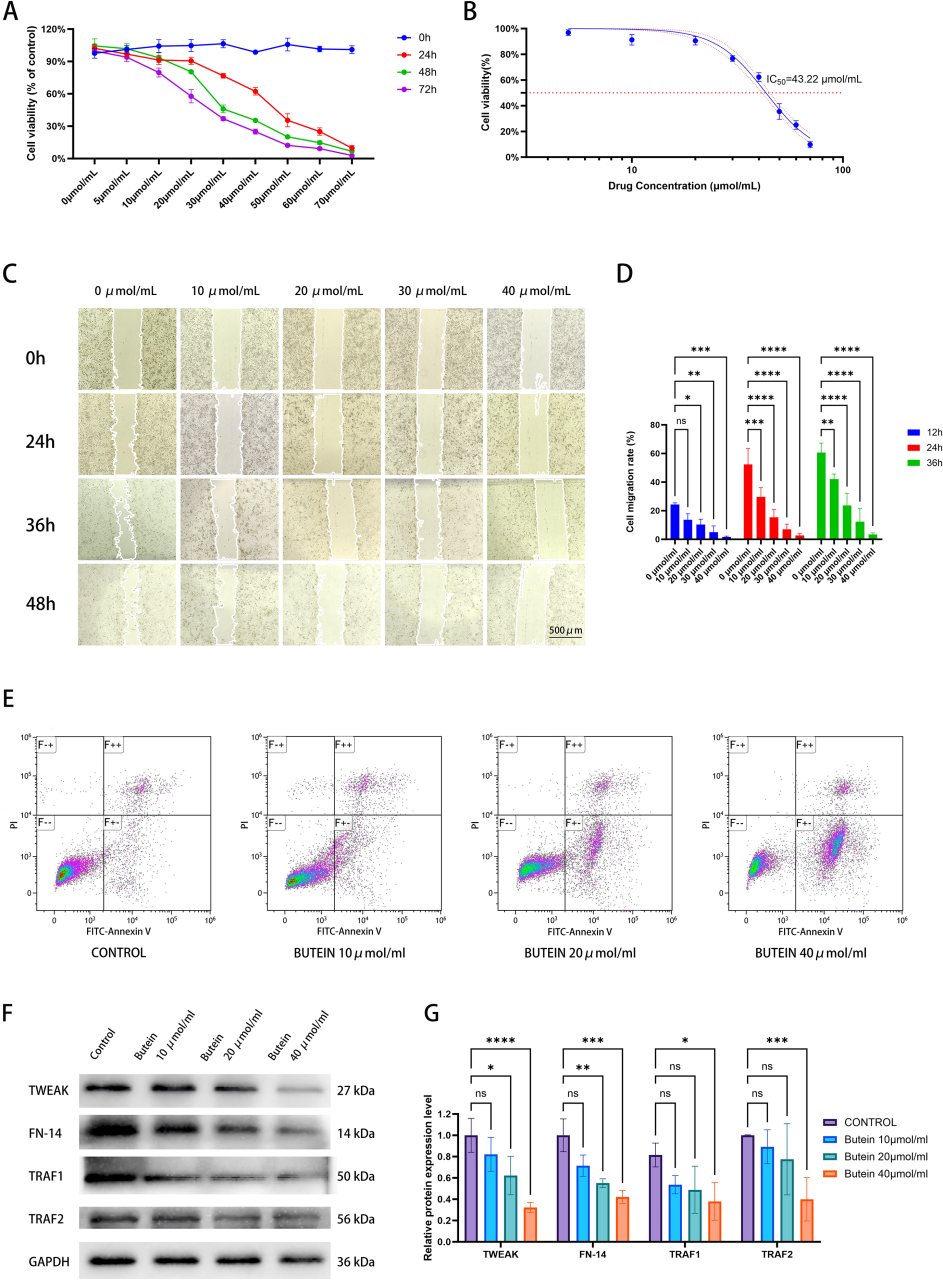
Butein inhibits A431 cell proliferation and migration while promoting apoptosis. **(A)** cell viability was assessed using the CCK-8 assay (n = 6 wells per concentration per experiment, three independent biological replicates). **(B)** The IC50 curve was calculated by nonlinear regression, with the 95% confidence interval indicated by the shaded area between dashed lines. **(C, D)** migration ability was evaluated by scratch assay (n = 3 independent experiments, three random fields per experiment). The wound area at 0 h and 48 h was quantified using ImageJ. Scale bar = 500 μm. Statistical analysis: two-way ANOVA (time × drug concentration), *p < 0.05, **p < 0.01, ***p<0.0005, ****p<0.0001. **(E)** apoptosis rate was determined by flow cytometry (n = 3 independent experiments, 10,000 cells collected per sample). Annexin V⁺PI⁻ indicates early apoptosis, while Annexin V⁺PI⁺ represents late apoptosis. Data are presented as mean±SD from three independent experiments. **(F)** Representative Western blot images (representative of three independent replicates). The loading amount was 10 μg per lane. GAPDH served as the internal reference, and molecular weights (kDa) are indicated on the right side of the bands. **(G)** protein quantification by band densitometry analysis (n=3 independent cell lysate samples, each quantified in three technical replicates). Comparisons with the control group were performed using an unpaired t-test, *p < 0.05, **p < 0.01, ***p<0.0005, ****p<0.0001.

To investigate the tumor-suppressive effect of Butein on cSCC, we first cultured the human cSCC cell line A431 cells and then treated them with different concentrations of Butein. Experimental data showed that Butein significantly inhibited the proliferation of A431 cells, with an IC_50_ value of 43 μmol/ml determined by the CCK-8 assay (**Figure 2B**). Based on this value, the concentrations for the low-, medium-, and high-dose groups were set at 10 μmol/ml, 20 μmol/ml, and 40 μmol/ml, respectively (**Figure 2A**). Subsequently, a scratch assay was performed to assess the effect of varying Butein concentrations on the migration ability of A431 cells. The results indicated that Butein markedly reduced the migration ability of A431 cells, and when the concentration reached 40 μmol/ml, the migration of A431 cells was significantly suppressed. (**Figures 2C, D**). Based on the data on cell proliferation inhibition, we selected a 24-hour treatment period as the optimal time frame for the apoptosis assay in A431 cells. The cells were treated with 10, 20, and 40 μmol/ml of Butein for 24 hours, and apoptosis induced by Butein was assessed using the FITC Annexin V/PI method. Our results demonstrated that Butein significantly increased the apoptosis rate in a dose-dependent manner (**Figure 2E**).

### 3.4 Butein reduces the expression of TWEAK, FN14, TRAF1, and TRAF2 in A431 cells

To further verify the inhibitory effect of Butein on proteins involved in the TWEAK-FN14 signaling pathway, A431 cells were treated with different concentrations of Butein. Western blot analysis was then performed to examine the expression levels of TWEAK, FN14, TRAF1, and TRAF2 proteins in A431 cells treated with 10 μmol/ml, 20 μmol/ml, and 40 μmol/ml of Butein, respectively. Western blot analysis revealed that Butein treatment downregulated the expression of TWEAK, FN14, TRAF1, and TRAF2 proteins in A431 cells compared with the control group (**Figures 2F, G**).

### 3.5 Butein inhibits the growth of cSCC in mice

**Figure 3:**
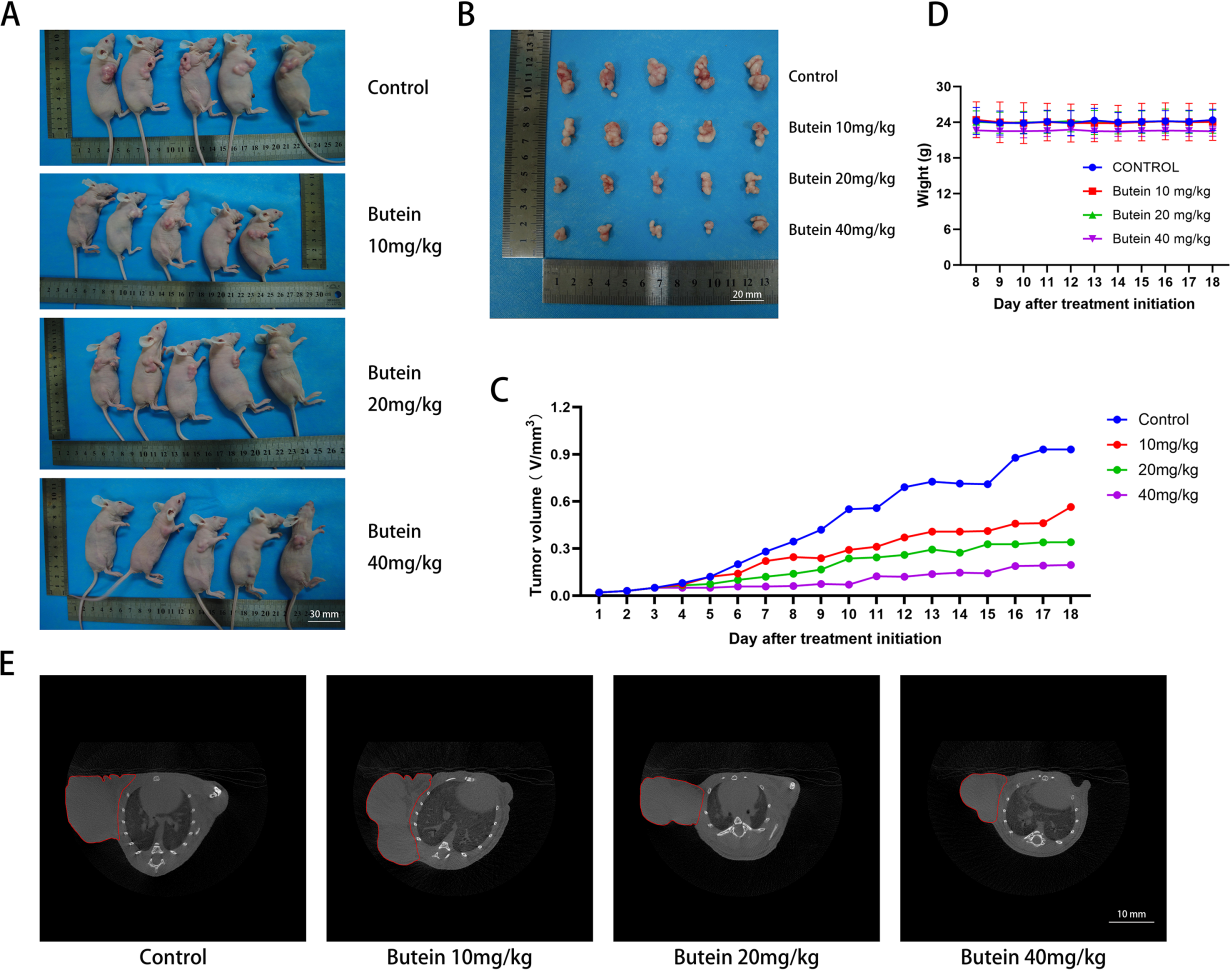
Butein inhibits cSCC xenograft tumor growth in Balb/c-nu mice. **(A)** Representative photograph of mice at the experimental endpoint. Scale bar = 30 mm. **(B)** Representative photograph of tumors at the experimental endpoint. Scale bar = 20 mm. **(C)** Tumor Volume Growth Curve (n = 5 mice per group, initial tumor volume ~50 mm³). Tumor volume was calculated as V = length × width² × 0.5. Data are presented as mean ± SEM. **(D)** Body Weight Change Curve (n = 5 mice per group). A body weight loss of <10% was considered tolerable. Note: Serum Biochemistry and Organ Histological Evaluation were not performed in this study; the conclusion on “Preliminary Safety” was based solely on body weight (see Section 4.5 for details). **(E)** Representative CT image of the tumor site in mice: the red-outlined area indicates the Tumor Tissue. Scale bar = 10 mm.

To further investigate the inhibitory effect of Butein on cSCC *in vivo*, a human cSCC xenograft model was established in Balb/c-nu mice. After successful tumor establishment, mice were treated with three different doses of Butein. The animal experiment data similarly demonstrated that the tumor size in Butein-treated mice was significantly smaller than that in the control group, indicating that Butein suppressed the growth rate of cSCC (**Figures 3A–C, E**). The tumor volume inhibition rates in the low-, medium-, and high-dose Butein groups were 39.21%, 63.44%, and 79.05%, respectively. Meanwhile, no significant changes in mouse body beight were observed among the four groups, suggesting that Butein exhibited low toxicity in mice (**Figure 3D**). It should be emphasized that serum biochemistry and organ histological evaluation were not performed in this study; therefore, the conclusion of “low toxicity” is based solely on body weight and gross appearance, representing only a signal of preliminary safety.A formal toxicity assessment requires the inclusion of hepatic and renal function data as well as histopathological evaluation, which will be the focus of our subsequent studies.

### 3.6 Butein reduces the expression of TWEAK and FN14 in tumor tissue

**Figure 4:**
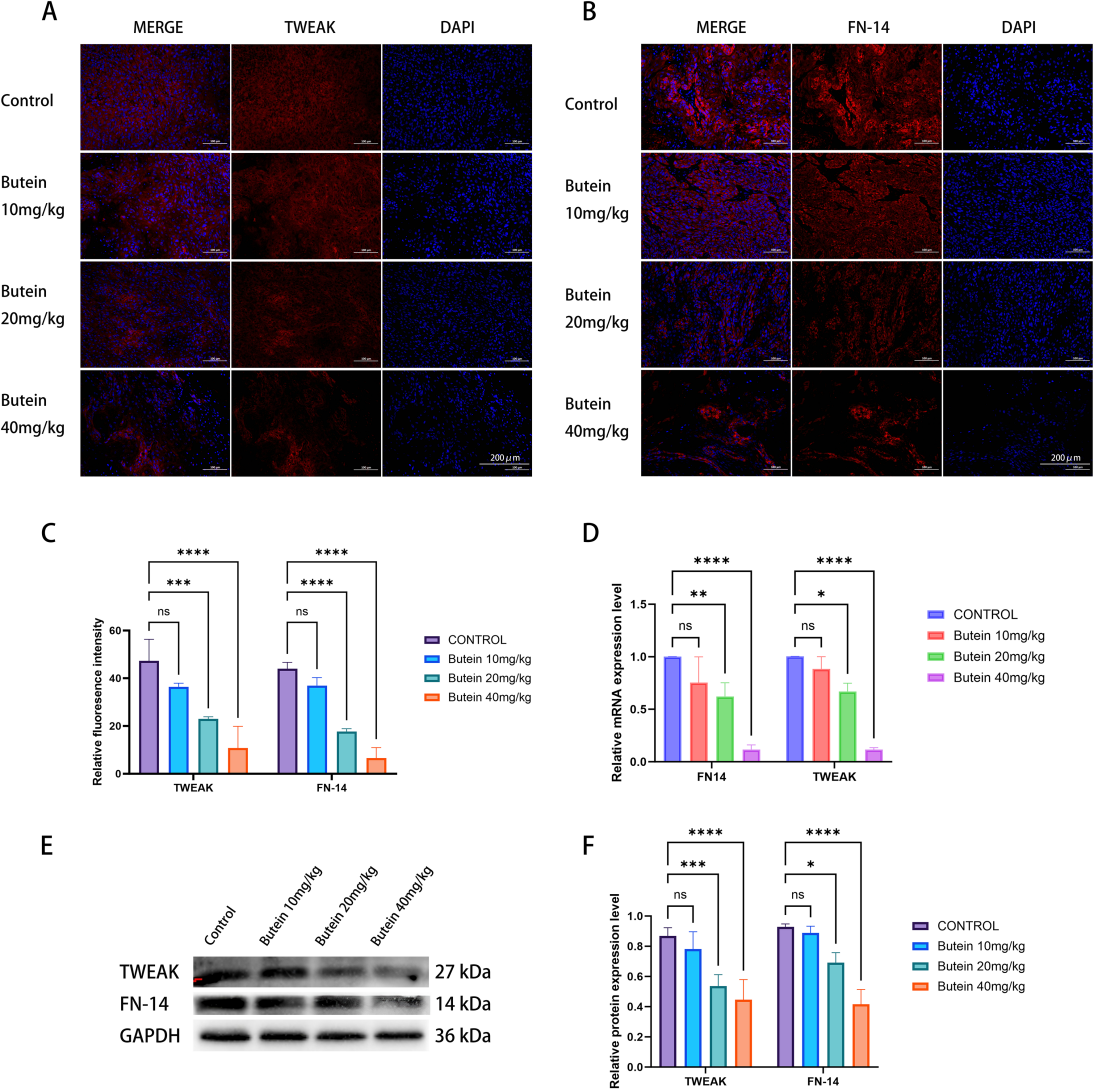
Butein reduces TWEAK/FN14 expression in Tumor Tissue. **(A, B)** Representative immunofluorescence images (n = 5 mice per group, three random sections per tumor). TWEAK (red), FN14 (red), and DAPI (blue). Scale bar = 200 μm. **(C)** Quantification of fluorescence intensity. Data are presented as mean±SD from five mice. Statistical analysis was performed using one-way ANOVA followed by Dunnett’s test versus the control group, ***p<0.0005, ****p<0.0001. Quantification was conducted using the “Measure” function of ImageJ with a constant ROI area. **(D)** RT-qPCR analysis of mRNA levels (n = 3 mice per group, two technical replicates per tumor). Relative expression was calculated using the ΔΔCT method, with GAPDH as the internal reference.*p < 0.05, **p < 0.01, ****p<0.0001 **(E)** Representative Western blot images (from three independent Tumor Tissue samples).loading amount: 10 μg per lane. molecular weights are indicated on the right side of the bands. **(F)** protein quantification and band densitometry analysis (n = 3 independent cell lysate samples, each quantified in 3 technical replicates). Statistical comparison with the control group was performed using an unpaired t-test, *p < 0.05, ***p<0.0005, ****p<0.0001.

Based on our previous studies, we further investigated whether Butein inhibits cSCC through the TWEAK/FN14 signaling pathway. Immunofluorescence (IF) results showed that, compared with the control group, the positive expression levels of TWEAK and its transmembrane receptor FN14 were significantly decreased in tumor tissue following Butein treatment (**Figures 4A–C**). Western blot analysis revealed that Butein administration downregulated the protein expression of TWEAK and FN14 in tumor tissue (**Figures 4E, F**). Consistently, RT-qPCR results confirmed that Butein reduced the mRNA expression levels of TWEAK and FN14 in tumor tissue (**Figure 4D**).

### 3.7 Butein reduces the expression of TRAF1 and TRAF2 in tumor tissue

**Figure 5:**
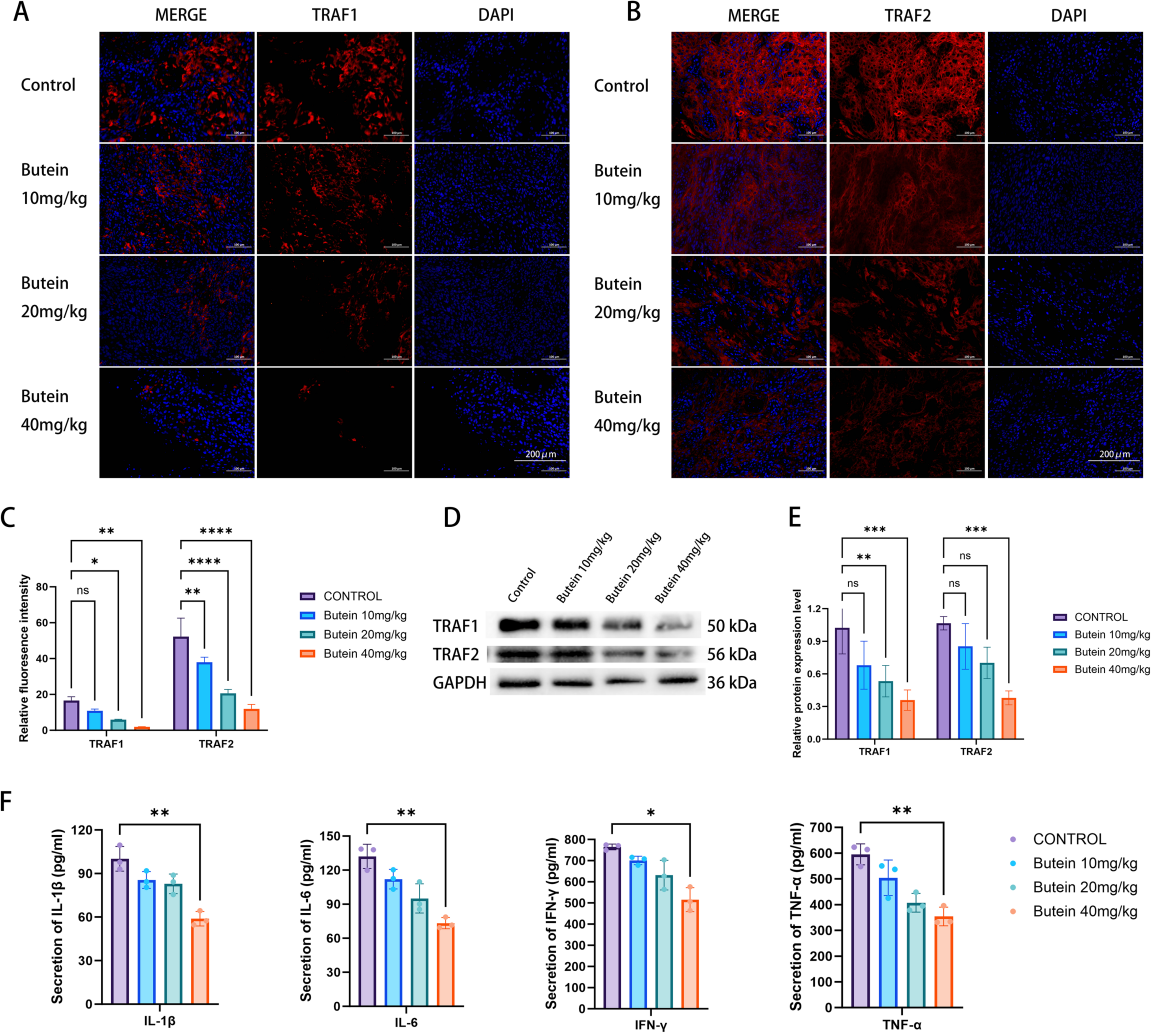
Butein downregulates TRAF1/2 and reduces serum inflammatory cytokines. **(A, B)** Representative immunofluorescence images (n = 5 mice per group, three random tumor sections per mouse). TRAF1 (red), TRAF2 (red), and DAPI (blue). Scale bar = 200 μm (indicated in the lower right corner of the merged images). **(C)** Quantification of fluorescence intensity. Data are presented as mean ± SD from five mice. Statistical analysis was performed using one-way ANOVA followed by Dunnett’s test, *p < 0.05, **p < 0.01, *** p<0.0005, **** p<0.0001. Quantification was conducted using the “Measure” function in ImageJ with a constant ROI area. **(D)** Representative Western blot images (from three independent Tumor Tissue samples). loading amount: 10 μg per lane. molecular weights are indicated to the right of the bands. **(E)** protein quantification by band densitometry analysis (n = 3 independent cell lysate samples, each quantified in three technical replicates). Statistical comparisons with the control group were performed using an unpaired t-test, **p < 0.01, *** p<0.0005, **** p<0.0001. **(F)** Serum ELISA (n = 5 mice per group, five technical replicates per sample). One-way ANOVA followed by LSD test was used for comparison with the control group,*p < 0.05, **p < 0.01. Error bars represent mean ± SD.

Subsequently, we examined whether Butein affects the expression levels of the downstream proteins TRAF1 and TRAF2 in the TWEAK/FN14 signaling pathway within tumor tissue. The results showed that the positive expression of both TRAF1 and TRAF2 was decreased following Butein treatment, with a more pronounced reduction observed at a Butein dose of 40 mg/kg (**Figures 5A–C**). Similarly, as the concentration of Butein increased, the protein expression levels of TRAF1 and TRAF2 in tumor tissue declined correspondingly (**Figures 5D, E**).

### 3.8 Butein decreases the levels of NF-κB-related inflammatory factors in mouse serum

Finally, we assessed the expression of inflammatory factors IL-1β, IL-6, IFN-γ, and TNF-α in mouse serum. The serum ELISA results indicated that the expression levels of IL-1β, IL-6, IFN-γ, and TNF-α were significantly lower in the experimental group compared with the control group (**Figure 5F**). Given that these four cytokines are typical transcriptional products of the NF-κB signaling pathway, this result is consistent with the inhibitory effect of the TWEAK-FN14 axis. However, since this assay did not include phenotypic analysis of *in situ* immune cells within the tumor tissue, it cannot comprehensively characterize the heterogeneity of the TME.

## 4 Discussion

### 4.1 Butein as a Potential antitumor drug for cSCC

This study provides comprehensive evidence for the antitumor effects of Butein against cSCC. The results demonstrate that Butein significantly inhibits the proliferation and migration of A431 cells—a classical cSCC cell line—in both *in vitro* and *in vivo* settings, while promoting apoptosis in a dose-dependent manner. These findings are consistent with previous studies showing that Butein exhibits potent antitumor activity against various cancers, including lung cancer, liver cancer, and osteosarcoma. The half-maximal inhibitory concentration (IC_50_) of Butein in A431 cells was determined to be 43 μmol/ml, indicating that it exerts strong efficacy at relatively low concentrations. This suggests that Butein holds promise as a potent candidate for the development of novel therapeutic strategies for cSCC.

### 4.2 The regulation of the TWEAK-FN14 pathway by Butein is highly correlated with its tumor-suppressive phenotype

This study revealed that the antitumor effects of Butein exhibit a “significant dose-dependent synchronous change” with the inhibition of the TWEAK-FN14-TRAF1/2 axis. *In vitro*, the IC_50_ (43μM) precisely corresponded to the concentration that reduced TWEAK protein expression by 50%. *In vivo*, administration of 40 mg/kg Butein resulted in a 79% reduction in tumor volume and completely blocked the nuclear translocation of TRAF2. Experimental data further confirmed that Butein markedly downregulated the protein and mRNA expression levels of TWEAK, FN14, TRAF1, TRAF2, and TRAIL-R3 in cSCC tumor tissue. These findings suggest that Butein may inhibit downstream signaling by interfering with the TWEAK-FN14 axis, thereby suppressing tumor growth and survival. The strong spatiotemporal correlation observed strongly indicates that this pathway may mediate the effects of Butein.However, we acknowledge that the current evidence is correlative rather than causal. Although the docking analysis showed that Butein binds directly to TWEAK with a binding energy of −6.9 kcal/mol, and *in vitro* experiments excluded off-target effects associated with cytotoxicity (apoptosis rate <30%), two possibilities remain. The first is the “primary-effect hypothesis,” which posits that Butein primarily targets the TWEAK-FN14 axis, with downstream NF-κB inhibition constituting the core mechanism of its antitumor activity. The second is the “synergistic hypothesis,” suggesting that inhibition of the TWEAK-FN14 pathway represents one component of Butein’s multitarget effects, acting in concert with the STAT3 and MAPK pathways.

### 4.3 Experimental approaches required to establish causality

To determine whether the TWEAK-FN14 axis serves as a necessary and sufficient mediator of Butein’s antitumor effects against cSCC, the following experiments are required:

First, a genetic epistasis experiment should be conducted by constructing A431 cell lines that overexpress TWEAK or constitutively activate NF-κB, in order to verify whether these manipulations can reverse the proliferation inhibition and apoptosis induction effects of Butein.If the phenotype is rescued, it would demonstrate that the pathway is necessary. Next, endogenous FN14 should be knocked down using siRNA to examine whether Butein loses its effect. If Butein shows no activity in FN14^-^/^-^cells, its specificity would be further confirmed. Second, pharmacological epistasis experiments: A431 cells are pretreated with a TWEAK agonist (rhTWEAK, 100 ng/mL) followed by Butein administration, to determine whether the agonist can counteract the effect of Butein. Third, pathway-specific transcriptomic analysis: RNA-seq is performed on A431 cells treated with Butein, focusing on the enrichment changes of the TWEAK-NF-κB signature gene set (e.g., IL-6, CXCL10, BCL-XL). If only the TWEAK-related gene set is significantly downregulated while the TNF-α pathway remains unaffected, the causal inference would be strengthened.

### 4.4 Effects on the tumor microenvironment and inflammatory factors

This study observed that Butein markedly reduced the serum levels of four NF-κB-dependent inflammatory factors, consistent with an overall improvement in systemic inflammatory status. These cytokines play critical roles in tumor progression, angiogenesis, and immune evasion. The significant downregulation of these cytokines following Butein treatment suggests that Butein may exert anti-inflammatory and anti-angiogenic effects, thereby further enhancing its antitumor activity. However, this finding does not constitute a comprehensive characterization of the TME. A thorough analysis of the TME should encompass the immune cell infiltration profile, stromal cell interactions, and the secretome landscape. Regarding the first aspect, the TWEAK-FN14 axis primarily regulates the polarization of TAMs (F4/80^+^CD86^+^) toward the M1 phenotype, the exhaustion of CD8^+^ T cells (PD-1^+^TIM-3^+^), and the expansion of Tregs (Foxp3^+^). This study did not assess these markers by immunofluorescence or flow cytometry. From the second perspective, CAFs (α-SMA^+^) amplify TWEAK signaling through the secretion of IL-6, forming a feed-forward loop. The absence of *in situ* α-SMA staining represents a gap in this study. Finally, the analysis of only four cytokines is insufficient to capture the heterogeneity of the TME. Multiplex cytokine detection platforms (such as the Mouse Cytokine 20-plex) can simultaneously quantify chemokine networks including CXCL10, CCL2, and IL-10. The true contribution of this study lies in establishing, for the first time, a “Butein-TWEAK-FN14-serum inflammation” correlation axis, providing an entry point for TME research rather than a complete depiction. Direct evidence of Butein’s causal effects on the TME requires additional *in situ* immunophenotypic analyses, which constitute a clear direction for future work.

### 4.5 Clinical significance and limitations

The findings of this study hold significant implications for the clinical management of cSCC. Given the rising incidence of cSCC and the limitations of current therapeutic options, Butein offers a novel therapeutic perspective for cSCC treatment. Its multi-pathway targeting capacity and modulatory effects on the tumor microenvironment suggest that Butein may serve as a potential monotherapy or be used in combination with existing treatments to enhance therapeutic efficacy. Future research should focus on conducting clinical trials to evaluate the safety and efficacy of Butein in patients with cSCC. In addition, further mechanistic studies are warranted to comprehensively elucidate the downstream effects of Butein on the TWEAK-FN14 pathway and its interactions with other signaling pathways involved in cSCC progression.The dose range of 10–40 mg/kg used in this study is well supported by preclinical evidence. Notably, the intraperitoneal bioavailability of Butein (~40%) is substantially higher than that of oral administration (<10%) (22), which explains why the doses applied in this study were lower than those typically used in clinical practice. Based on body surface area conversion, a mouse dose of 20 mg/kg corresponds to an approximate human equivalent dose of 1.6 mg/kg, or about 100 mg per day for an adult, which falls within the conventional dosage range of natural product supplements.This study has certain limitations. First, formal PK/PD modeling was not performed. Future studies should determine the time-course concentrations of Butein in plasma and tumor tissue using LC-MS/MS and establish a PK/PD model to further refine the dosing interval.In addition, topical administration of Butein to the skin (e.g., via nanoemulsion cream) may further reduce systemic exposure by directly targeting the lesion, representing an important direction for future translational research. Second, this study did not perform molecular docking Re-docking validation (RMSD) or Molecular dynamics simulation, nor did it include direct binding assays such as SPR, ITC, or CETSA. Consequently, dynamic binding stability could not be demonstrated. The correlation between predicted binding energy and phenotypic data (r = 0.88) constitutes indirect evidence and does not exclude the possibility of indirect regulation. Future work will include CETSA to assess TWEAK thermal stability changes, Biotin-Butein pull-down combined with mass spectrometry to identify Interacting proteins, and a 200-ns MD simulation to verify conformational changes in TWEAK-TRAF2. Third, the characterization of the TME in this study is limited, as only four serum cytokines were measured. No *in situ* immune phenotyping (e.g., spatial distribution of CD8^+^ T cells and CD68^+^ macrophages), single-cell sequencing, or spatial transcriptomics was conducted, preventing a comprehensive elucidation of the mechanisms by which Butein remodels the immunosuppressive TME. Fourth, the major limitation of this study is that the safety of Butein was assessed solely based on body weight changes, without providing serum biochemistry (ALT/AST/BUN/CRE) or organ histopathology (H&E) evidence. Although existing literature supports that Butein at comparable doses (10–40 mg/kg) has an LD_50_ > 500 mg/kg and causes no hepatic or renal injury (23, 24), such data cannot substitute for safety evaluation within this study itself. Prior to clinical translation, repeated-dose toxicity studies and topical dermal safety assessments must be completed. A 28-day preliminary toxicity study has been prioritized in our project plan, and the results will be reported in subsequent publications. Fifth, this study employed only the A431 cell line and did not include validation in other cSCC cell lines such as SCC-12, which limits the generalizability of the conclusions.

### 4.6 Barriers to clinical translation

The development of Butein as a therapeutic agent for cSCC must overcome three major translational gaps. The first concerns metabolic stability and bioavailability. Butein, a polyphenolic compound, undergoes rapid phase II hepatic metabolism (glucuronidation and sulfation) following oral administration, resulting in extremely low systemic exposure (t_1_/_2_ ≈ 1.5 h, F < 5%) (25). In this study, intraperitoneal injection was employed to bypass the first-pass effect; however, clinical translation requires the development of a topical transdermal delivery system. Nevertheless, Butein exhibits poor aqueous solubility (<50 μg/mL) and a relatively high molecular weight (272.25 Da), which limit its skin permeability. Nanoemulsions, liposomes, or microneedle arrays may serve as potential solutions (26, 27). The second concerns its applicability to human cSCC.The A431 cell line used in this study was derived from immunodeficient nude mice and therefore cannot recapitulate the role of immune checkpoints (PD-1/PD-L1) within the TME. Recent clinical studies have demonstrated that high expression of TWEAK-FN14 in human cSCC is associated with PD-L1 positivity and resistance to immunotherapy (16). Whether Butein can be combined with PD-1 inhibitors such as cemiplimab requires validation in humanized mouse models. Third, the safety window. The LD_50_ of Butein (>500 mg/kg) indicates low toxicity; however, long-term topical application may induce photosensitivity or contact dermatitis. This study observed only a 2-week dosing period, and subchronic toxicity (3 months) as well as carcinogenicity assessments have not yet been conducted. The FDA requires topical formulations of natural products to undergo skin sensitization testing (OECD TG 429), which represents a critical prerequisite for advancing to preclinical evaluation.

### 4.7 Comparison and positioning relative to current frontline therapies for cSCC

In recent years, the therapeutic landscape of cSCC has undergone a revolutionary transformation due to the advent of immune checkpoint inhibitors (ICIs) and targeted agents, necessitating a clear definition of Butein’s role within this context. First, regarding its complementarity with immunotherapy: Cemiplimab (a PD-1 antibody) has been approved for metastatic cSCC, achieving an objective response rate (ORR) of 47% (28). Its mechanism involves restoring T-cell function, whereas Butein acts by suppressing the TWEAK-FN14 axis to reduce immunosuppressive cytokines (IL-6 and TNF-α); thus, their mechanisms are orthogonal. Theoretically, their combination could yield synergistic effects, with Butein purifying the TME and thereby enhancing the efficacy of ICIs. This concept underlies the core strategy of current clinical trials investigating TWEAK inhibitors (such as RO7121930). Keytruda (Pembrolizumab) has shown efficacy in PD-L1-high cSCC, yet the rate of primary resistance remains as high as 30% (29).Butein’s potential to downregulate PD-L1 (via STAT3 inhibition) may partially overcome therapeutic resistance. Second, in contrast to targeted agents: EGFR inhibitors (erlotinib and afatinib) are effective against EGFR-mutant cSCC but their use is limited by dermatologic toxicity (acneiform rash) (30). As a natural compound, Butein exhibits lower skin irritation and does not depend on EGFR mutation status, thereby benefiting a broader patient population. Hedgehog pathway inhibitors (Vismodegib) are effective only in basal cell carcinoma (BCC), as Smoothened mutations are rare in cSCC, rendering them ineffective (31). Butein demonstrates superior target universality. Third, Butein possesses unique advantages by simultaneously inhibiting TWEAK-FN14 (immune modulation), STAT3 (proliferation), and Nrf2 (oxidative stress), aligning with the multifactorial pathogenesis of cSCC. Compared with ICIs, which cost over $150,000 annually, Butein, as a naturally derived compound, is more accessible and suitable for use in resource-limited settings or as adjuvant therapy after surgery. From the perspective of future clinical positioning, Butein is most likely to serve as a second-line combination therapy or an alternative option for patients intolerant to ICIs, rather than as a first-line monotherapy. The current priority is to complete GLP toxicology studies and phase I dose-escalation trials.

## Data Availability

The datasets presented in this study can be found in online repositories. The names of the repository/repositories and accession number(s) can be found in the article/**Supplementary Material**.
