# Supplementary material for: Mechanism of action of Butein in cutaneous squamous cell carcinoma through regulation of the TWEAK-FN14 signaling pathway
Source: Front Oncol. 2025 Dec 11;15:1725848. doi: 10.3389/fonc.2025.1725848 (PMC12738174; doi:10.3389/fonc.2025.1725848)
Supplement: Supplementary file 1 [file Table1.docx]

*Table S1. Primer sequenees for RT-gPCR*

|  | | ***Gene*** | ***Primer sequence(5’-3’)*** |
| --- | --- | --- | --- |
| ***1*** | *mTWEAK-Forward* | | *CCTCCTGCTGGTCGTGGTC* |
|  | *mTWEAK-Reverse* | | *ATCCTGGCTTTCCTCTGTCTGG* |
| ***2*** | *mFN14-Forward* | | *GGGATTCGGCTTGGTGTTGATG* |
|  | *mFN14-Reverse* | | *GCCAAAACCAGGACCAGACTAAG* |
